# Role of Receptor Activity Modifying Protein 1 in Function of the Calcium Sensing Receptor in the Human TT Thyroid Carcinoma Cell Line

**DOI:** 10.1371/journal.pone.0085237

**Published:** 2014-01-13

**Authors:** Aditya J. Desai, David J. Roberts, Gareth O. Richards, Timothy M. Skerry

**Affiliations:** The Mellanby Centre for Bone Research, Department of Human Metabolism, University of Sheffield, Sheffield, United Kingdom; Cleveland Clinic Lerner Research Institute, United States of America

## Abstract

The Calcium Sensing Receptor (CaSR) plays a role in calcium homeostasis by sensing minute changes in serum Ca^2+^ and modulating secretion of calciotropic hormones. It has been shown in transfected cells that accessory proteins known as Receptor Activity Modifying Proteins (RAMPs), specifically RAMPs 1 and 3, are required for cell-surface trafficking of the CaSR. These effects have only been demonstrated in transfected cells, so their physiological relevance is unclear. Here we explored CaSR/RAMP interactions in detail, and showed that in thyroid human carcinoma cells, RAMP1 is required for trafficking of the CaSR. Furthermore, we show that normal RAMP1 function is required for intracellular responses to ligands. Specifically, to confirm earlier studies with tagged constructs, and to provide the additional benefit of quantitative stoichiometric analysis, we used fluorescence resonance energy transfer to show equal abilities of RAMP1 and 3 to chaperone CaSR to the cell surface, though RAMP3 interacted more efficiently with the receptor. Furthermore, a higher fraction of RAMP3 than RAMP1 was observed in CaSR-complexes on the cell-surface, suggesting different ratios of RAMPs to CaSR. In order to determine relevance of these findings in an endogenous expression system we assessed the effect of RAMP1 siRNA knock-down in medullary thyroid carcinoma TT cells, (which express RAMP1, but not RAMP3 constitutively) and measured a significant 50% attenuation of signalling in response to CaSR ligands Cinacalcet and neomycin. Blockade of RAMP1 using specific antibodies induced a concentration-dependent reduction in CaSR-mediated signalling in response to Cinacalcet in TT cells, suggesting a novel functional role for RAMP1 in regulation of CaSR signalling in addition to its known role in receptor trafficking. These data provide evidence that RAMPs traffic the CaSR as higher-level oligomers and play a role in CaSR signalling even after cell surface localisation has occurred.

## Introduction

The G-protein coupled receptor (GPCR) family is the largest family of cell-surface receptors in mammals and is involved in numerous vital functions such as taste, odour, memory, response to light, and the action of hormones and neurotransmitters [1]. The Calcium Sensing Receptor (CaSR) is a family C GPCR that binds calcium principally and plays an essential role in systemic calcium homeostasis [2]. The CaSR is involved in regulation of parathyroid hormone (PTH) secretion from parathyroid chief cells [2], and calcitonin secretion from thyroid parafollicular C-cells [3]. PTH has complex physiological functions, increasing serum calcium by enhancing bone resorption and (through its promotion of activation of Vitamin D) absorption of calcium from the gut and reducing renal calcium excretion [4]. Transient high PTH levels have the opposite effect, mediating increased bone formation by an as yet poorly understood pharmacological mechanism [5]. In contrast, calcitonin inhibits the function of bone-resorbing osteoclasts and reduces urinary calcium excretion[6]. The importance of CaSR in calcium homeostasis is emphasized by the pathological conditions of calcium homeostasis caused by inactivating and activating CaSR mutations such as Familial Hypocalciuric Hypercalcaemia (FHH), Neonatal Severe Hyperparathyroidism (NSHPT) [7], [8]; and Autosomal Dominant Hypocalcaemia (ADH) [9]. The CaSR is a promiscuous receptor which binds a variety of natural and synthetic ligands such as the cations, Ca^2+^, Mg^2+^, Gd^3+^, Zn^2+^, Ni^2+^ [2], [10], [11]; the polyamines, spermine and spermidine [12]; aminoglycoside antibiotics such as neomycin [13], phenylalkylamine derivatives including calcimimetics (allosteric activators) [14], [15] and calcilytics (antagonists) [16]. Following activation of the CaSR, downstream signalling is complex and cell-type dependent. CaSR is shown to couple to more than one G-protein subtype (G_q_, G_i_, G_s_, G_12/13_ [17]–[20]) and to activate MAPKs such as ERK1/2 [21], [22] that elicit different actions based stimulus and the cell type. It has been demonstrated that in transfected cells, the CaSR is unable to traffic to the cell membrane alone [23], [24], and that two members of a family of type-1 trans-membrane proteins known as Receptor Activity Modifying Proteins (RAMPs 1 and 3) interact with CaSR and facilitate its localization at the cell surface.

The RAMPs were discovered in attempts to clone the receptor for calcitonin gene-related peptide (CGRP) [25]. It was discovered that the “CGRP receptor” was a heteromer of the calcitonin-like receptor (CLR) and RAMP1. The CLR forms an adrenomedullin receptor with either RAMPs 2 or 3 [25], while RAMP association of the calcitonin receptor forms 3 variants of a receptor for Amylin [26]. Since then, the roles of RAMPs in regulation of ligand selectivity and trafficking have become well-established in several high profile studies. The role for RAMPs in trafficking was detected first in association with the CLR [25], which like the CaSR, requires a RAMP for expression at the cell surface [23]. Other receptors are known to traffic with RAMPs (including the PTH, glucagon, VIP [27], and secretin receptors [28]), but these are not obligate relationships and those receptors do not require RAMPs for surface expression. In the case of VPAC1/VIP receptor, association of RAMP2 leads to an increase in inositol phosphate hydrolysis [27], showing a direct role of RAMP in modifying the signalling of the receptor. So far, the only known family C GPCR partner of RAMPs is the CaSR [23], and no other functions except for cell surface trafficking of the receptor have been identified.

The aims of the current study were: 1) to acquire more detailed information about the CaSR/RAMP interactions during trafficking and cell surface presentation, and 2) to determine whether CaSR/RAMP interactions in cells naturally expressing endogenous levels have functional relevance.

Here we show, using sensitized FRET and FRET-based stoichiometry, new data regarding interactions of RAMPs 1 and 3 with the CaSR. Additionally, we show that in human medullary thyroid carcinoma cells (TT), a significant reduction in the signalling of the CaSR when RAMP1 is knocked down or blocked with a RAMP1 antibody.

## Methods

### Cell lines

COS-7 and TT cell lines (ATCC) were maintained in T-175 cm^2^ flasks (Nunclon, Thermo scientific) in complete DMEM and F-12K medium (GIBCO, Paisley) respectively, at 37°C in 5% CO_2_ incubator. The complete medium contained 10% heat inactivated Fetal Calf Serum (FCS, GIBCO Paisley), 1 mM Sodium Pyruvate (Sigma-Aldrich) and 1% penicillin and streptomycin (Sigma-Aldrich). Thereafter, the media were changed twice a week until the cells were confluent. Cells used in experiments were in their exponential growth phase.

### Preparation of constructs for FRET and COS-7 cell transfection

Citrine or Cerulean cDNAs were engineered into a pcDNA3.1 vector (Invitrogen) between the Not1 and Xho1 restriction enzyme sites. RAMPs and CaSR were engineered into pcDNA3.1 Cerulean and Citrine vectors respectively excluding their stop codons, between the Kpn1 and Not1, and HindIII and Not1 restriction enzyme sites, so that the fluorophores were present at the C-terminal of RAMP/CaSR. As a negative control, pcDNA 3.1 containing Citrine alone were co-transfected with a pcDNA3.1 RAMP Cerulean vector. As a positive control, we created a pcDNA3.1 vector containing a Cerulean cDNA fusion construct followed by 18 amino acid linker sequence and then Citrine cDNA.

COS-7 cells were grown to confluency and harvested using trypsin/EDTA (Sigma), washed with PBS, and resuspended in electroporation buffer (composition [mM] 20 HEPES, 135 KCl, 2 MgCl_2_, 2 ATP, 5 glutathione, 0.5% Ficoll 400 adjusted to pH 7.6 using KOH) at a concentration of ∼3.5– 4 million cells into 4 mm gap electroporation cuvettes (York Biosciences, UK) and the required concentration of DNA was added (10µg receptor, 15µg RAMP constructs). The cells were then electroporated at 0.25 kV and 960 µF using a Gene Pulser (Biorad) and cultured for 72 hr in 35 mm glass-bottom plated (Ibidi, München) after which they were fixed with 4% PFA and mounted with Mowiol.

### FRET imaging

Images were captured using a Zeiss Plan apo 63×/1.4 oil immersion lens on a Zeiss LSM 510 inverted laser scanning confocal fluorescence microscope fitted with an argon laser at room temperature. Confocal images of the fluorescent proteins were acquired using an argon laser together with an HFT458/514 nm dichroic, a NFT515 nm beam splitter, pin hole set to 496µm, detector gain 550 and individually as a separate channel under the following conditions: Cerulean was excited using the 458 nm laser line with a 100% laser intensity and a band pass BP480–520 emission filter; Citrine was excited using the 514 nm laser line attenuated to 20% laser intensity and a band pass BP535–590 emission filter; FRET was excited using the 458 nm laser line with a 100% laser intensity and a BP480–520 emission filter. All fluorescence channels were scanned and the collected together, line by line with a mean of 1.

Cerulean and Citrine fluorescence bleed-through into the FRET channel were calculated using FRET and co-localization analyser plugin for ImageJ [29]. NFRET calculations for FRET efficiency for sensitized emission were done using pixel-by-pixel analysis by PixFRET plugin for ImageJ [30]. The threshold for pixel intensity to be included in analysis was set to 1.5 times the background intensity. The following equation was used to calculate FRET efficiency:
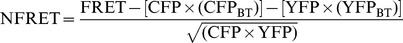



BT =  bleed through

The correction factors calculated were: β (proportionality constant relating donor fluorescence detected at the acceptor emission relative to that detected at the donor emission): 0.31, α (proportionality constant relating acceptor fluorescence at the acceptor excitation to the donor excitation): 0.126, γ (ratio of the extinction coefficient of the acceptor to the donor at the donor excitation): 0.3, ξ (proportionality constant relating the sensitized acceptor emission to the decrease in donor fluorescence due to FRET): 0.2.

Cell surface FRET was separated from whole cell FRET by constructing a series of 50-pixel diameter dots around the cell surface of the raw acceptor image using the selection tool of Image J. Each dot was taken as a ROI and the combined ROIs for each image were used to calculate mean membrane NFRET and stoichiometry values. All FRET-based stoichiometric analysis was performed as previously described [31] using ImageJ software.

### Knock-down of RAMP1 mRNA expression in TT cells using siRNA

In order to knock-down RAMP1 mRNA expression, TT cells were transfected with RAMP1 siRNA or negative control scrambled (sc) siRNA using electroporation. The RAMP1 and sc siRNA sequences were the same as used successfully by Bouschet *et al* [23]. Approximately 1.5 million TT cells were transfected with 1.5µg of RAMP1 or sc siRNA in 0.4 ml final volume at 960µF and 0.22 kV. After electroporation, TT cells were cultured in 24-well clear bottom plates in complete F-12K medium for 72 hr, before using them for further experiments.

To confirm efficient knock-down of RAMP1 expression, RNA was extracted from the experimental and control transfected cells using Trizol (Invitrogen) 72 hr after transfection and cDNA was synthesized from equal amounts of RNA using high-capacity RNA to cDNA kit (Applied biosystems). Gene expression was measured using Taqman® probes for quantitative PCR using the ABi 7900HT sequence detection system (Applied biosystems). The cytoskeletal protein beta actin (Actβ) was used as an endogenous control for normalizing the expression of target genes. 2^−ΔCt^ method was used for relative expression analysis.

### Immunocytochemistry

TT cells were seeded on 15×15 mm glass coverslips (Menzel-Glaser). The cells were washed twice with PBS and fixed for 10 min at room temperature using 4% PFA (Sigma Aldrich), 48 hr post transfection. Cells were incubated with 10% rabbit serum (Vector labs) and 0.5% BSA (w/v) for 30 min at room temperature. Following this, primary antibodies (RAMP1 sc-8850- Santacruz biotech or Goat IgG- Vector labs) were incubated overnight at 4°C in 1% rabbit serum with 0.5% BSA (w/v) in PBS at 1∶50 v/v dilution of the 0.2µg/µl stock concentration. Coverslips were washed with 0.5% BSA (w/v) in PBS and then incubated with secondary antibody (Rabbit anti goat IgG conjugated with FITC, DAKO) for 45 min in dark at room temperature in 1% rabbit serum with 0.5% BSA in PBS at 1∶400 dilution of 2.5µg/µl stock concentration. Following the incubation, the coverslips were washed with 0.5% BSA (w/v) in PBS and incubated with DAPI counterstain at 1∶5000 dilution of 5 mg/ml stock concentration for 3 min followed by further three washes with 0.5% BSA (w/v) in PBS. The coverslips were drained to remove excess liquid and mounted on a clean glass slide (VWR international) using Prolong Gold (Invitrogen) and stored in dark at room temperature overnight before imaging.

HCX PL FLUOTAR L 40.0×0.60 dry objective on an Inverted widefield fluorescence microscope LeicaDMI4000B was used to capture images at 8-bit resolution and 1×1 binning at room temperature. Separate channels were set each for FITC (exposure 1.5 sec, gain 3.5, filter-L5), DAPI (exposure 150 ms, gain 3.0, filter A4) to capture RAMP1 or control IgG staining and nuclear staining respectively.

### Western blotting

Sub confluent cultures of transiently transfected COS-7 cells or TT cells were harvested using 2 mm glass beads in ice cold PBS. The cells were then spun and lysed on ice using a Dounce homogeniser, and the resulting lysate was spun at 40,000 g for 40 minutes at 4°C. The pellet containing membranes was then resuspended in buffer containing 100 mM NaCl, 50 mM HEPES, 5 mM MgCl_2_, adjusted to pH 7.4 with KOH [32]. For western blots of COS-7 cells to determine the localization of CaSR expression transfected with the different RAMPs, both total cell lysates and membrane samples were analysed.

Immunoblotting was performed by separating the components of 10–15µg of CaSR+RAMP transfected COS-7 or TT cell membrane on 8% or 12% SDS-PAGE gel to probe for CaSR or RAMP expression respectively. The gel was electrophoresed at 90 V and proteins were transferred to Hybond-P polyvinylidene chloride (PVDF) membrane (Amersham) at 100 V for 80 min. Proteins of interest were detected by first incubating with primary antibodies (RAMP1, sc-8850 Santacruz biotechnology 1/300 v/v dilution from 0.2µg/µl stock concentration, the CaSR ab-19347, Abcam was diluted 1/500 v/v from 1µg/µl stock concentration.) overnight at 4°C, followed by a HRP conjugated secondary antibody (anti goat or anti mouse HRP conjugated IgG immunoglobulin, Sigma Aldrich at 1∶10,000 dilution) for 1 hour. Chemiluminescence was detected using detecting photographic film (Hyperfilm™ ECL, Amersham).

### Intracellular calcium imaging

TT cells were seeded at a density of 100,000 cells per well in a 24-well clear-bottom plate (Costar, Corning) for two days before intracellular calcium assays. Media were removed and cells were washed twice with PBS and loaded for 45 min at 37°C with 500µl of physiological salt solution (buffer) containing: (contents [mM]: 2 CaCl_2,_ 100 NaCl, 5.4 KCl, 1.2 MgSO_4_, 5.5 Glucose, 6 NaHCO_3_, 1.2 Na_2_HPO_4_, 20 HEPES, pH 7.4), 5µM Fluo-4AM dye (5 mM stock in DMSO) and 2.5 mM water soluble probenecid. After incubation cells were washed three times with the buffer and further incubated with 500µl of buffer containing 2 mM CaCl_2_ for 45 min at 37°C. During this incubation, the cells were treated with antibodies (RAMP1 goat polyclonal antibody, sc-8850, Santacruz biotech; Goat control IgG, Vector labs) according to the requirement of the experiment. After 45 min, the buffer was replaced with 360µl of physiological salt solution containing 1.5 mM CaCl_2_ when using Cinacalcet, or containing 2 mM CaCl_2_, when using Neomycin as an agonist. The cells were imaged using an HCX PL FLUOTAR 10.0×0.30 dry objective on an Inverted wide field fluorescence microscope (Leica AF6000 Time Lapse) at 37°C (single channel fluorescence image using the L5 filter, with exposure of 1 sec, at gain and intensity of 5). Images were taken at 12-bit resolution at every 1.2 sec with first 35 frames recorded as baseline after which 40µl of 10X solution of agonist was added carefully and images were recorded for further 3 min. Images from each well were exported in.tiff format and were analysed using ImageJ software. A time-dependant response curve was plotted using GraphPad Prism version 5.00 for Windows (GraphPad Software, San Diego California USA, www.graphpad.com) and peak value of response for each cell was calculated using AUC function. The peak value of response for each cell was then calculated as percentage change from baseline fluorescence and expressed in the graph as percentage above baseline.

### Fluorescence-Activated Cell sorting (FACS)

COS-7 cells were transfected as mentioned before and 48 hr later were washed with PBS, harvested using non-enzymatic dissociation and fixed using 4% PFA for 15 min and resuspended in FACS buffer (PBS, 1% FBS). Cells were then treated with 5% FBS, washed and incubated with 1.2µg mouse CaSR antibody (abcam, Cambridge, USA) per 10^6^ cells for 1 hr at room temperature, and subsequently with secondary antibody conjugated with FITC (DAKO, Carpinteria, USA) (1µg/10^6^ cells) for 30 min. As negative controls cells were incubated with mouse IgG control, as well as with the secondary antibody only. From each sample, ten thousand cells were analysed using a flow cytometer (FACSCalibur, Becton Dickinson, San Jose, CA) after excluding cell debris.

### Statistical analysis

All data shown are mean± SEM. The graphs were plotted in and the statistical analysis was performed using GraphPad Prism version 5.00 for Windows (GraphPad Software, San Diego California USA, www.graphpad.com). The statistical tests performed for each specific experiment is shown in the relevant results section. Normalcy test was performed on each set of data using D’Agostino & Pearson omnibus normality test using Graphpad prism 5 software. The precise number of cells and replicate experiments is detailed in the relevant figure legends.

## Results

### Cell membrane FRET-based stoichiometric analysis of the interaction of CaSR with RAMPs

The interaction of RAMPs with CaSR in transfected COS-7 cells was confirmed using FRET. Sensitized emission FRET was used to compare the efficiency of interaction of RAMPs with CaSR, and FRET-based stoichiometry to calculate the fractions of RAMP and CaSR in the FRET complexes on the cell membrane. As a negative control in order to measure the background levels of FRET- a combination of Citrine only, along with different RAMP-Cerulean constructs were used.

**Figure 1:**
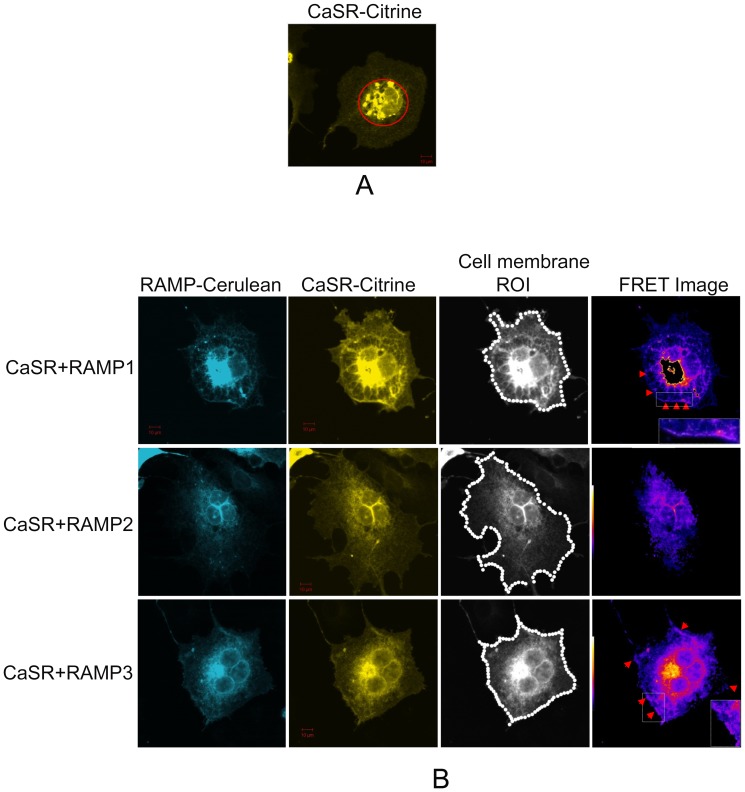
Spatial localization of CaSR and RAMPs in COS-7 cells. (A) COS-7 cell transfected with CaSR-citrine alone showing intracellular localisation of CaSR (red circle) in the absence of RAMP expression. (B) COS-7 cells co-transfected with RAMP-cerulean (left column) and CaSR-citrine (column 2) were imaged by confocal microscopy 48 hr post transfection. 50 pixel dot ROI was manually drawn around the cell membrane of the CaSR-citrine image to measure cell-surface FRET (column 3). Scale bar 10µm. Red arrows on the FRET images (right column) indicate areas of co-localization between CaSR and RAMP on the cell-surface, which are shown magnified in the insets.

When COS-7 cells were transfected with CaSR alone, majority of the receptor was localized within the peri-nuclear region (figure 1A). However, when either RAMP1-Cer or RAMP3-Cer were co-transfected with the CaSR, distinct regions of co-localization of FRET complexes were seen ranging from the peri-nuclear region and cytoplasm, towards the cell membrane (figure 1B). Red arrows on the FRET figures indicate cell-surface FRET complexes of CaSR+RAMP1/3 (figure 1B and high power insets). In case of RAMP2-Cerulean, co-localization with CaSR could be seen in the perinuclear region only with no detectable cell-surface co-localization (figure 1B).

**Figure 2:**
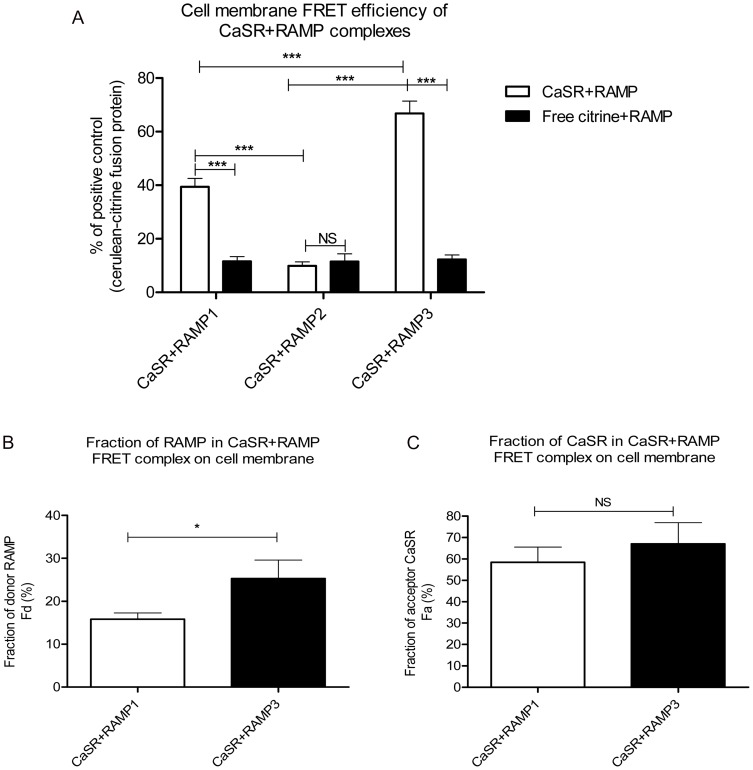
Cell-surface FRET efficiencies of CaSR+RAMPs and fraction of receptor components involved in FRET complex. (A) Cell-surface FRET efficiencies of individual RAMPs with CaSR compared among themselves and also with respective negative control Citrine alone+RAMP-Cerulean. Data are expressed as a percentage of a positive control comprising cells expressing a Citrine-Cerulean fusion protein. *** p<0.001 (2-way ANOVA, Bonferroni post-test) ***p<0.001 (Kruskal-Wallis test, Dunn's multiple comparison test) (B) and (C) Stoichiometric analysis of fraction of donor RAMP (Fd) and acceptor CaSR (Fa) present in FRET complex on the cell-surface, respectively.* p<0.05 Mann Whitney test. The graph represents data from three independent experiments.

Intensity values from the donor, acceptor and FRET images were obtained from cell membrane regions using a series of 50 pixel-dots to define regions of interest (ROI) on the acceptor image (figure 1B third column). Using these values, cell-surface NFRET values and stoichiometric calculations were performed for the CaSR (figure 2A,B and C and Table 1). The cell-surface NFRET values for CaSR+RAMP1/3 combinations were significantly higher than their corresponding negative controls, and CaSR+RAMP2 cell-surface FRET complex (figure 2A; Table 1). NFRET between CaSR and RAMP2 was not significantly different from the corresponding negative control (RAMP2-Cerulean and Citrine alone). CaSR+RAMP3 cell-surface FRET efficiency was found to be significantly higher by ∼1.6 fold compared to CaSR+RAMP1 (figure 2A, Table 1). As shown by the R value (ratio of total CaSR to RAMP expression on the cell surface), there was an equal excess of RAMP expression compared with CaSR in both CaSR+RAMP1/3 transfected cells. Of this, the fraction of total RAMP1 that was at the cell surface, involved in cell-surface FRET complex was 16±1.4%, whereas for RAMP3 the proportion on the surface was 26.0±4.3% (figure 2B and Table 1). So the fraction of RAMP3 on the cell surface was ∼1.6 fold higher than RAMP1 in FRET complex with CaSR, which was significant. The fraction of CaSR present in the FRET complex on the cell-surface was 58.4±7.1% (with RAMP1) and 67.00±10.0% (with RAMP3) (figure 2C and Table 1); out of its total population. There was no statistically significant difference between the fraction of CaSR in RAMP1 and RAMP3 cell-surface FRET complexes. This means that both RAMP1 and 3 exhibited equal efficiencies in trafficking CaSR to the cell surface, however there was a higher fraction of RAMP3 (donor) in the FRET complex on the cell surface, consequently causing an increased FRET efficiency, compared to RAMP1.

**Table 1:** Mean and SEM values of cell membrane FRET efficiency and fraction of receptor components involved in FRET between the CaSR and RAMPs.

CaSR+RAMP	NFRET % of positive control	Fraction of acceptor CaSR Fa (%)	Fraction of donor RAMP Fd(%)	R	Calculated ratio CaSR:RAMP
**CaSR+RAMP1**	^a^40.0±3.4***^ vs b^	58.4±7.1	16.0 ±1.4	0.6±0.09	2.19: 1
**CaSR+RAMP2**	^b^9.9±1.5	-	-	0.5 ±0.11	-
**CaSR+RAMP3**	65.3±4.4 ***^ vs a,b^	67.00±10.0	26.0±4.3*	0.6±0.2	1.5: 1

Significant difference in the NFRET value was observed between CaSR+RAMP1 and 3 vs. CaSR+RAMP2, and between CaSR+RAMP1 vs. CaSR +RAMP3 (***p<0.0001, Kruskal-Wallis test, Dunn's multiple comparison test). Significant differences were also observed in the fraction of RAMP3 and RAMP1 donor (Fd) in cell-surface FRET complex (*p<0.05, Mann Whitney test). There was no significant difference between fraction of CaSR acceptor in the FRET complex between RAMPs 1 and 3 (Fa). The table displays combined data of three independent experiments.

### Cell surface expression of non-tagged CaSR

**Figure 3:**
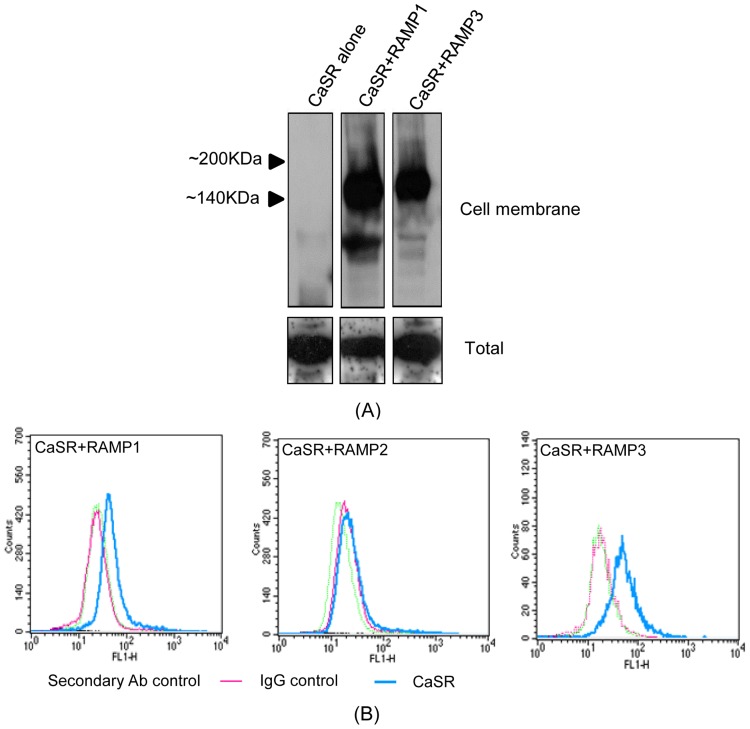
Cell surface expression of non-tagged CaSR. (A) Western blot of membrane and total cell lysate preparations from COS-7 cells transfected with CaSR alone (lane 1), CaSR and RAMP1 (lane 2), and CaSR and RAMP3 (lane 3), incubated with antibody to the CaSR, demonstrating the ability of RAMPs 1 and 3 to traffic the CaSR to the cell surface. (B) FACS analyses of non-permeabilised COS-7 cells expressing CaSR with either RAMPs 1, 2 or 3 showing shift from the IgG control in number of cells with surface fluorescence in RAMP1 and RAMP3 expressing cells, but not RAMP2 expressing cells.

In membrane preparations from COS-7 cells expressing CaSR alone, no CaSR protein could be detected by western blotting (figure 3A, lane 1). In contrast, when CaSR was expressed with either RAMP1 or RAMP3, in the western blots, there were clear bands between 140 and 200 kDa corresponding with the predicted size of the CaSR (Figure 3A, lanes 2 and 3 respectively). As a control, CaSR was detected in all the three conditions in samples prepared from COS-7 cell lysates, indicating total protein expression.

We confirmed these results using FACS on the same (non-permeabilised) COS-7 cells. In cells expressing the CaSR with RAMP1, we measured clearly increased levels of surface fluorescence compared with binding of isotype control antibodies, indicating RAMP1-mediated cell surface expression of CaSR (figure 3B left panel). In cells expressing CaSR and RAMP2, which we know from the FRET studies to not be an effective trafficking partner, there was no shift with CaSR compared with isotype control antibodies, indicating no surface expression (figure 3B, centre panel). In cells expressing CaSR and RAMP3, we observed surface expression greater than the isotype controls (figure 3B right panel), and greater than in cells expressing CaSR with RAMP1.

### Attenuation of CaSR signalling by modulation of RAMP1 expression in TT cells

**Figure 4:**
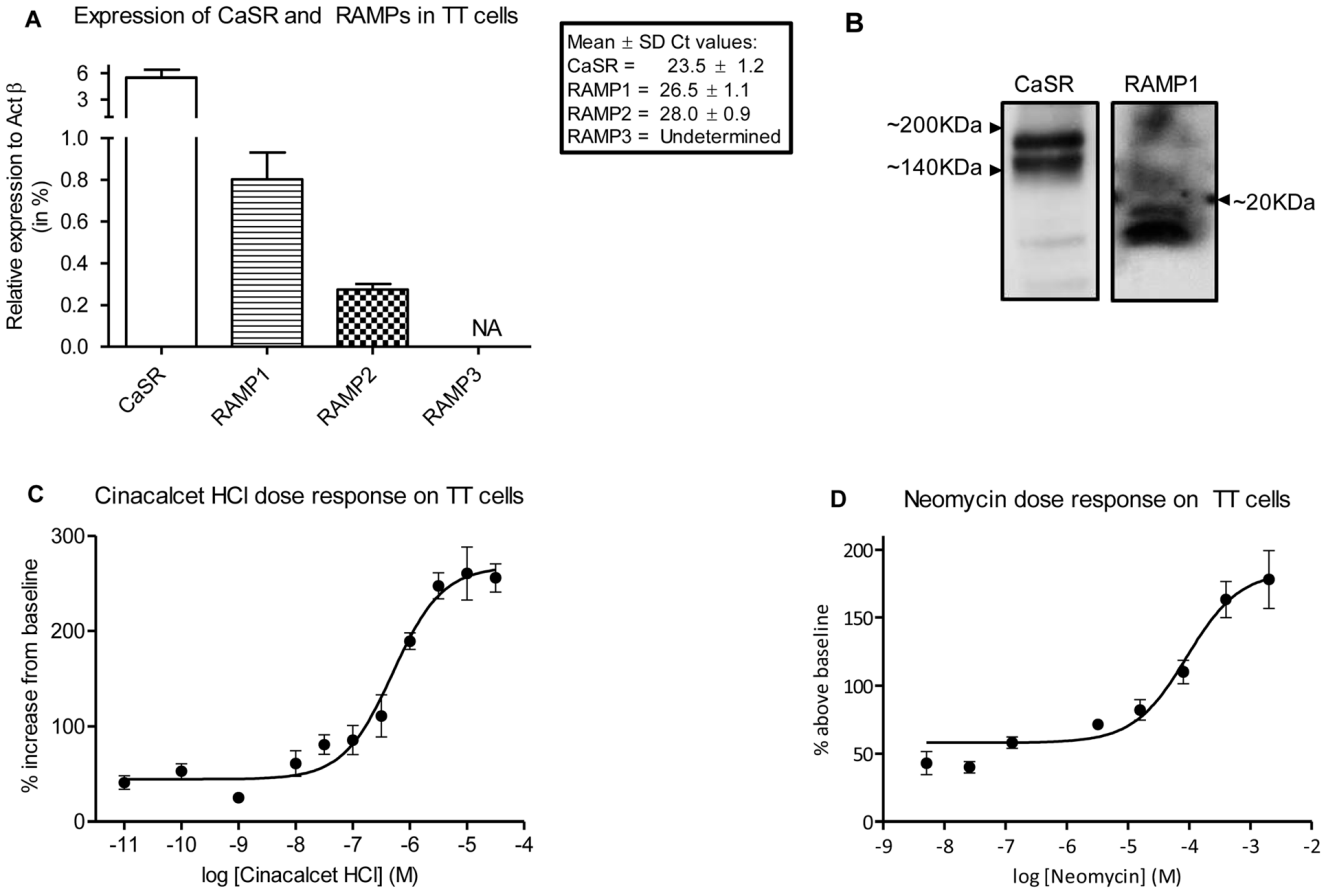
Expression of CaSR and RAMP1 in TT cells. (A) mRNA expression levels of CaSR and RAMPs in control TT cells. (B) Representative western blot showing protein expression of CaSR and RAMP1 in TT cell membranes. (C) Concentration-dependent Cinacalcet-induced increase in intracellular calcium release in TT cells in presence of 1.5 mM CaCl_2_ (Ec_50_ 503±1.29 nM). The data are combined from three independent experiments with a total of n = 50 cells analysed at -4.5, -5, -5.5, -6 and -11 M; 20 cells analysed at −6.5, −7, −7.5 and −8 M; and 30 cells analysed for −9 and −10 M concentrations. (D) Concentration-dependent Neomycin-induced response in presence of 2 mM CaCl_2_ (Ec_50_ 91±1.45 µM). The data are combined from three independent experiments with a total of n =  43, 48, 45, 46, 88, 74, 23 and 29 cells analysed per concentration respectively, going from high to low doses.

To explore the role of RAMP1 in CaSR signalling, we used TT cells that express mRNA and protein endogenously for CaSR, RAMP1 and RAMP2 but not RAMP3 (figure 4A and 4B). To confirm the functional activity of the CaSR in TT cells, increases in the intracellular calcium levels were measured using live cell imaging. We obtained concentration-response curves for the allosteric modulator of the CaSR, Cinacalcet in presence of 1.5 mM CaCl_2_ (Ec_50_ 503±1.29 nM), and the agonist Neomycin in presence of 2 mM CaCl_2_ (Ec_50_ 91±1.45 µM) (figure 4C and 4D) by recording images for ∼ 3.5 min at every concentration.

**Figure 5:**
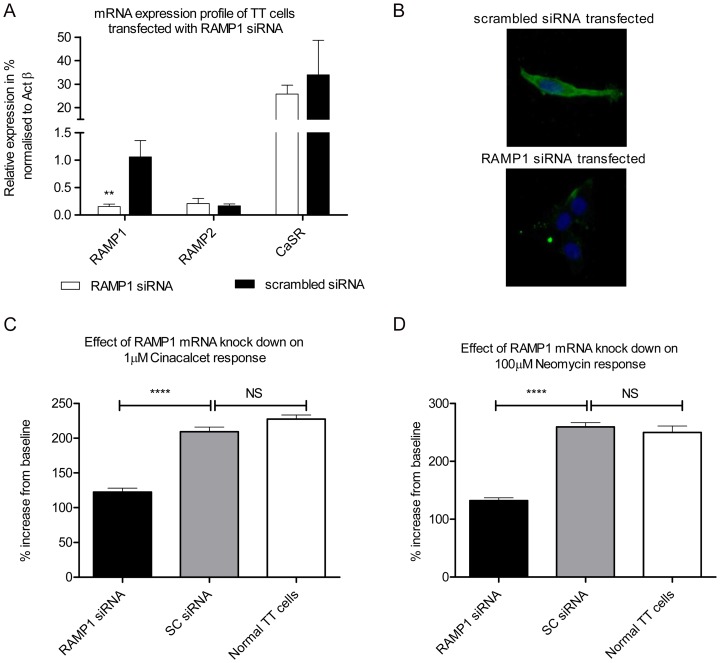
Effect of RAMP1 mRNA knockdown on TT cells. (A) mRNA expression levels of RAMP1, RAMP2 and CaSR in TT cells transfected with RAMP1 or scrambled siRNA, 72 hr post-transfection expressed as fold change normalised to Actβ. (B) Representative images from immunofluorescent staining for RAMP1 expression in cells transfected with scrambled siRNA (top panel) and RAMP1 siRNA(bottom panel), 72 hr after transfection. (C) Intracellular calcium response of the RAMP1 siRNA cells to 1µM Cinacalcet in presence of 1.5 mM CaCl_2_ and (D) 100µM Neomycin in presence of 2 mM CaCl_2_ The responses were decreased by ∼42% and ∼50% respectively compared to scrambled siRNA transfected cells. The data are combined from five independent experiments with a total of 241, 231 and 154 cells analysed in (C), and 354, 354 and 118 cells analysed in (D) for knock-down, control and normal conditions respectively. **** p<0.0001 analysed by two-tailed Mann-Whitney test.

The functional responses to Cinacalcet were compared between TT cells, whose RAMP1 expression had been attenuated using siRNA and controls, transfected with an appropriate scrambled sequence. 72 hr after transfection, significant decrease in mRNA (by ∼80%) and protein expression levels of RAMP1 were observed in RAMP1 siRNA-transfected TT cells compared to the scrambled control (Figure 5A and B respectively). There was no effect of RAMP1 or scrambled siRNA transfection on the mRNA expression levels of RAMP2 or CaSR (Figure 5A). TT cells transfected with RAMP1 or control scrambled siRNA were treated with 1µM Cinacalcet in presence of 1.5 mM CaCl_2_ or 100µM Neomycin in presence of 2 mM CaCl_2_ and signalling was quantified by measuring increase in intracellular calcium using a live cell imaging system. The response to 1µM Cinacalcet was significantly attenuated by ∼42% in RAMP1 siRNA-transfected cells compared to the negative control (figure 5C). There was no difference between the signalling of the negative control and control non-transfected TT cells, excluding non-specific effects due to transfection. 100µM Neomycin, induced ∼50% less elevation in the intracellular calcium release in RAMP1 siRNA-transfected cells compared with both scrambled transfected and native controls (figure 5D).

### Attenuation in CaSR signalling by RAMP1 antibody in TT cells

**Figure 6:**
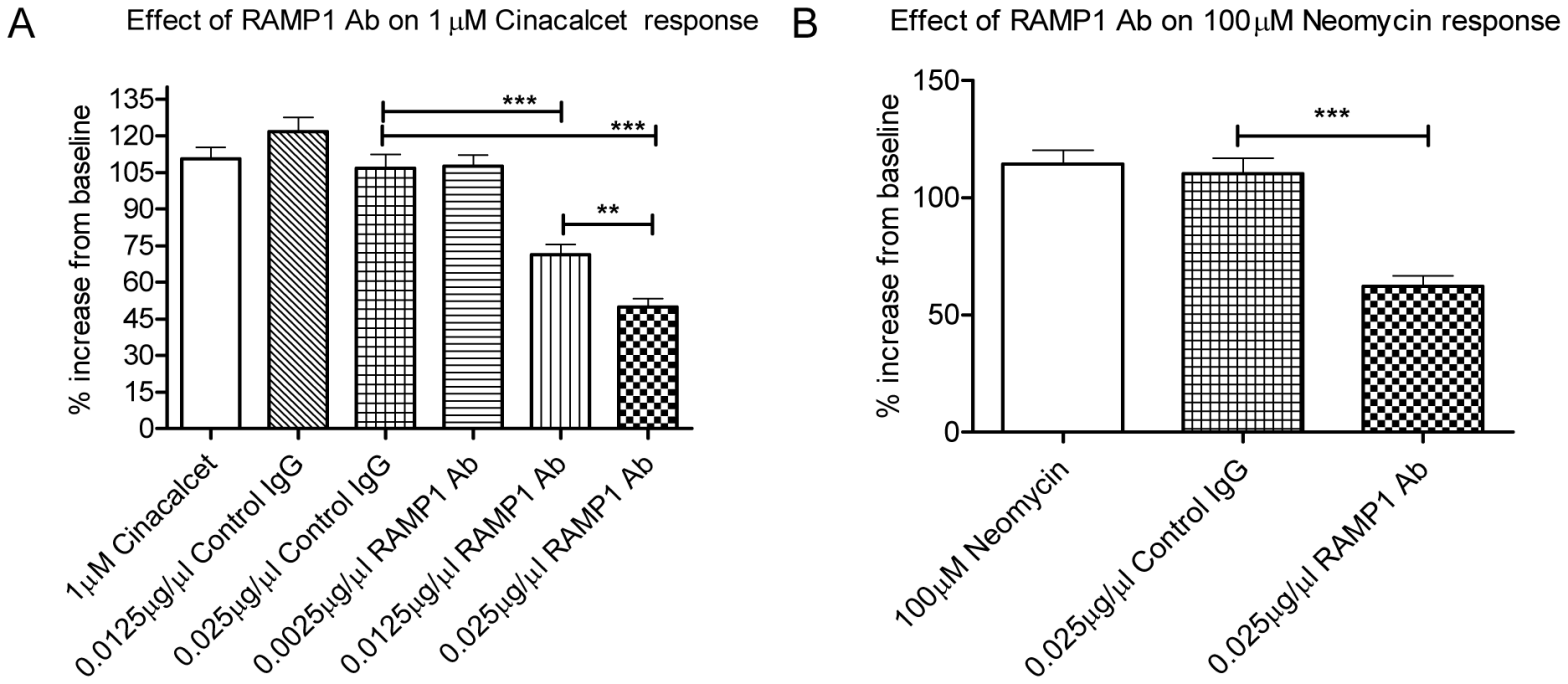
Attenuation of CaSR response in TT cells by RAMP1 antibodies. (A) Concentration-dependant decrease in Cinacalcet-induced intracellular calcium release in presence of 1.5 mM CaCl_2_, by RAMP1 antibodies in TT cells. Total cells analysed combined from five independent experiments are 272 (1µM Cinacalcet), 261 (0.0125µg/µl control IgG), 240 (0.025µg/µl control IgG), 252 (0.0025µg/µl RAMP1 Ab), 272 (0.0125µg/µl RAMP1 Ab) and 250 (0.025µg/µl RAMP1 Ab). (B) 0.025µg/µl RAMP1 antibody caused a significant inhibition of 100µM Neomycin response in presence of 2 mM CaCl_2_ compared to control IgG. Total cells analysed combined from five independent experiments are 192 (100µM Neomycin), 177 (0.025µg/µl control IgG) and 166 (0.025µg/µl RAMP1 Ab). *** p<0.001 determined by Kruskal-Wallis test, Dunn's multiple comparison post-test, ** p<0.01 two-tailed Mann-Whitney test.

TT cells were treated with a characterised commercial RAMP1 polyclonal antibody [33]–[35], which induced a concentration-dependent attenuation of intracellular calcium release in response to 1µM Cinacalcet in presence of 1.5 mM CaCl_2_ (figure 6A). The response to 1µM Cinacalcet was significantly attenuated by 0.0125µg/µl and 0.025µg/µl dose of RAMP1 antibody by 35% and 57% respectively (both p<0.001). The differences between the effect of 0.0125µg/µl and 0.025µg/µl RAMP1 antibody (21%) was statistically significant (p<0.01). It was also observed that 0.025µg/µl RAMP1 antibody reduced 100µM Neomycin response in presence of 2 mM CaCl_2_, significantly by ∼48% compared to Control IgG (p<0.001) (figure 4B).

## Discussion

RAMPs are promiscuous proteins that are shown to engender different receptor phenotypes to a few family B GPCRs [25], [27], [28], [36]. It was discovered that the association of the CaSR with RAMP 1 or 3 is essential for its cell-surface trafficking in transfected cells [23], [24]. In order to investigate further the consequences of this interaction, we hypothesized that RAMPs associate with the CaSR to form higher-order oligomeric complexes, and play a role in signalling of the CaSR. Particularly, this study has characterized additional functions of RAMP1 in complex with the CaSR, implying a two-tier regulation which comprises cell surface trafficking and signalling of the CaSR.

In this study we have used a series of well-established techniques to confirm and extend previous work in the area using transfected cells, and to determine the relevance of those findings in a more physiological setting of a cancer cell line. The FRET techniques we have used differ from established techniques only in our use of a membrane region of interest to compare total and cell surface interactions. This very simple analysis of FRET data could be useful in other research where proteins are trafficked to the cell surface from inside the cell. Our studies increase knowledge of the RAMP/CaSR interactions because we have analysed the stoichiometric relationships between the proteins involved, something which was not performed in earlier studies using tagged constructs. It should be emphasised that our results were obtained only by FRET, a recognised method to determine protein-protein interactions. Further confirmatory studies using different methods such as co-immunoprecipitation in both over-expressing and native cells could be used to provide additional support for our findings. Such techniques may have the ability to reveal shifts in the proportion of oligomers, dimers and monomers.

It could be suggested that the fluorophore-protein constructs may not behave identically to native proteins. However, we show using cells expressing untagged receptor constructs, that RAMPs 1 and 3 but not 2 are required for cell surface expression of the receptor by FACS and western blotting. In addition, FRET is well established, and our data are entirely consistent with studies using a different technique to show interactions of RAMPs 1&3 but not RAMP2 with the CaSR [23]. The other techniques we have used are well-established. Knockdown siRNA sequences are those used by others [23], and we demonstrate their effectiveness in reducing mRNA and protein expression. The effects of the knockdown and antibody treatments are disclosed using a well-established readout of elevation of intracellular calcium after treatment with pharmacological ligands.

FRET-based stoichiometric analysis in this study indicated that a unit of CaSR-complex on the cell surface with either RAMP consist of ∼1.6 times more RAMP3 than RAMP1. Recently the presence of high order oligomers of other GPCRs at cell surface has been reported [37]-[39]. However, it still remains to be understood how many units of CaSR and RAMPs heteromerize to form larger oligomeric complexes on the cell membrane. It is possible that such large oligomers could contain different RAMPs with the CaSR, but the complete functional consequences of such complex heteromeric structures are not understood yet. An additional level of regulation could be provided by the PDZ-binding motif which is present on RAMP3 [40], which can have effects on recycling and internalization of the CaSR, as already reported in the case of the adrenomedullin receptor-2 (CLR+RAMP3) [40], [41].

The demonstration that knockdown of RAMP1 expression attenuates CaSR signalling could be due to a decreased population of CaSR+RAMP1 complexes at the cell-surface. However, the ability of antibodies to attenuate the ability of CaSR ligands to induce elevation of intracellular calcium release points to an additional more complex role for RAMPs in CaSR signalling. There are several potential mechanisms for this. The physical presence of the antibody bound to the RAMP1 part of the RAMP1/CaSR complex could prevent normal ligand binding. Alternatively, the antibody may interfere with the interaction between the RAMP and CaSR so that they no longer form a functional heteromer. A third possibility is that antibody bound to the RAMP1 may affect the internalisation of the receptor complex to reduce the presence of functional receptor heteromers on the cell surface. It is also conceivable that antibody induces conformational changes in the receptor that normally follow ligand binding or coupling to the G-proteins of the CaSR so as to alter downstream signalling. It is unlikely, though still possible, that RAMP1 is directly involved in the formation of the ligand binding pocket, since the sites for binding of Ca^2+^ [42] (ECD), neomycin (ECD) [43], and the calcimimetic Cinacalcet (TM domain) [44], [45], have been mapped on the CaSR itself.

The physiological relevance of the findings remain to be confirmed in detail, and studies in cells from a range of species, and to measure calcitonin secretion in cells with altered RAMP expression would be valuable. The development of tissue-specific knockout mice for the RAMPs will also provide opportunities for greater insight into their roles in calcium homeostasis specifically and endocrinology generally.

In conclusion, this study has provided novel insights into the interaction of CaSR and RAMPs which point towards the presence of a higher oligomeric receptor-complex, with the possibility of more than one RAMP molecule per CaSR. Secondly, RAMP1 is essential for the cell surface trafficking of the CaSR in endogenously expressing cells. Finally, RAMP1 plays a role in the signalling of CaSR in TT cells that are at the cell surface, by an unknown mechanism. Therapeutic targeting of RAMP1 may be a method to modulate calcium homeostasis via the CaSR.
